# The experience of individuals following non-surgical management of Achilles tendon rupture in the United Kingdom – a qualitative study

**DOI:** 10.1371/journal.pone.0352761

**Published:** 2026-06-29

**Authors:** Samuel Briggs-Price, Tom Yates, Jitendra Mangwani, Maneesh Bhatia, Seth O’Neill

**Affiliations:** 1 University of Leicester, School of Healthcare, Leicester, United Kingdom; 2 Diabetes Research Centre, University of Leicester, Leicester, United Kingdom; 3 Orthopaedics, University Hospitals of Leicester, Leicester, United Kingdom; MountainView Hospital, UNITED STATES OF AMERICA

## Abstract

**Objectives:**

Despite extensive research on Achilles tendon ruptures (ATR), the lived experience of patients remains under explored. This study aimed to investigate the experiences of individuals managed non-surgically within the National Health Service (NHS).

**Design:**

Qualitative study using semi-structured, one-to-one interviews.

**Setting:**

NHS services in the United Kingdom providing non-surgical management for ATR.

**Participants:**

Fifteen patients (mean age 54.5 years) who had sustained an ATR and were managed non-surgically. Participants were recruited from a specialist ATR clinic.

**Methods:**

A stakeholder-informed topic guide was used to conduct semi-structured interviews. Interviews were audio-recorded, transcribed verbatim, and analysed using reflexive thematic analysis. Coding was undertaken independently by two researchers, with themes reviewed by the full research team. The study followed COREQ guidelines for qualitative reporting.

**Results:**

Three themes were identified: (1) The experience of injury and entering the healthcare system: ‘it felt as if my whole ankle had exploded’ participants described sudden injury onset, sometimes painless, and varied understanding of treatment options; (2) The experience of non-surgical immobilisation: ‘you’re being told so much in that first session…I just couldn’t remember’ boot immobilisation was valued for mobility but challenged comfort and self-management; and (3) The rehabilitation journey: ‘it happened so unexpectedly so it can happen again’ fear and uncertainty about rehabilitation and returning to sport were common, shaping participant’s rehabilitation experience.

**Conclusions:**

This study highlights how diverse ATR injury experiences, early management variability, and boot-related challenges impact recovery. Fear of re-rupture shapes rehabilitation and return to sport, emphasising the need for clearer guidance on boot use, weightbearing, and rehabilitation progression, alongside consistent healthcare professional support to optimise outcomes.

## Introduction

The Achilles tendon is the common tendon of the triceps surae complex, formed by the merging of the medial and lateral gastrocnemius and soleus muscles. Achilles tendon ruptures (ATR) most frequently occur approximately 6 cm proximal to the calcaneal insertion, an area where the triceps surae merges into a single tendon unit [1]. ATR often results from mechanisms such as pushing off the weight-bearing foot with the knee extended, unexpected dorsiflexion of the ankle, or violent dorsiflexion of a plantarflexed foot [2,3]. Despite advances in management, significant long-term functional deficits persist years after rupture, with enduring impairments in maximal strength, endurance, and gait parameters [4–6].

Much of the existing ATR literature has focused on functional immobilisation protocols, objective outcomes, or rehabilitation regimes, but there remains a lack of qualitative research exploring the lived experiences of patients, especially in non-surgical pathways. Previous qualitative investigations have primarily centred on surgically managed cohorts, highlighting patient preferences for surgery and satisfaction with outcomes despite initial frustrations [7,8]. However, these studies do not reflect the reality of care in the United Kingdom (UK), where non-surgical management is the primary approach [9]. Non-operative treatment typically involves plaster casts or functional boots to approximate tendon ends, but little is known about how patients experience these protocols in practice [10,11].

Existing qualitative studies have explored patient perspectives years after injury, introducing recall bias and overlooking how the acute response to ATR can shape rehabilitation behaviours [12]. Each healthcare system also presents unique challenges, although no study has examined how NHS-specific factors such as resource pressures and care pathways influence patients’ recovery journeys.

This study aimed to explore the initial 12 months of lived experiences of individuals with ATR treated non-surgically in the NHS, focusing on pain and function, psychological responses such as fear and confidence, interactions with healthcare services, experiences of immobilisation and rehabilitation, and attempts at returning to sport. Understanding these experiences is crucial to improving patient support, enhancing adherence to evidence-based protocols, and optimising recovery outcomes.

## Materials and methods

This study used a qualitative design, with reflexive thematic analysis of in-person, one-to-one, semi-structured interviews conducted with individuals who had experienced an ATR and were managed in the NHS. Interviews were completed between August and November 2023 at a UK University. Interviews were directed by a topic guide developed by the authorship team and stakeholders who had experienced ATR during the development stage of the study. These patient stakeholders contributed to the development and refinement of the topic guide by reviewing the relevance, clarity, and acceptability of proposed interview questions. Interviews were recorded using a digital voice recorder and transcribed verbatim. The topic guide is available in S1 File. All interviews were completed by the lead author (SBP) only, a male physiotherapist completing interviews as part of a PhD studentship. The interviewer was experienced at qualitative methodology. The interviewer was not involved in the clinical care of the participants. The lead author’s professional background as a physiotherapist and PhD researcher provided familiarity with ATR management and rehabilitation pathways, which supported the development of clinically relevant interview prompts. To support reflexivity, the lead author maintained a reflexive diary during data collection and analysis, and emerging codes and themes were discussed with the wider authorship team.

We adhered to the Consolidated Criteria for Reporting Qualitative Research (COREQ) guideline for reporting of qualitative research [13]. Ethical approval for the study was granted by London – Bromley Research Ethics Committee (22/PR/1720). All participants provided informed written consent prior to completing an interview.

### Participants

Purposive sampling was used to ensure a representative distribution of both sexes based on previous studies [14]. Participants had been diagnosed with an ATR and attended an NHS ATR clinic between August 2022 and June 2023. The NHS ATR clinic was the primary management site for ATRs across the county. The ATR clinic routinely uses the Leicester Achilles Management Protocol (LAMP) when managing acute ATRs. The recruitment strategy aimed to achieve data saturation, with a maximum of 20 participants. Following the recruitment of 10 participants, transcripts were analysed by authors SBP and SO to assess for data saturation, which was defined as the point during data analysis at which incoming data yielded little or no new information relevant to the study objectives [15]. Saturation was assessed during the recruitment period, after the initial 10 interviews had been completed and transcribed, and was reviewed alongside ongoing recruitment. Recruitment continued until data saturation was reached, or until the upper limit of 20 participants was met.

### Data analysis

Transcriptions were stored and analysed using NVivo software (version 12, QSR International, Doncaster, Australia). Reflexive thematic analysis described by Braun and Clarke (2019) was used to analyse transcripts [16]. The six stages of thematic analysis were used as an analysis framework; familiarising yourself with the data, generating initial codes, searching for themes, reviewing themes, defining and naming themes and producing the report. A reflexive diary was completed by the lead author (SBP).

Authors (SBP, SO) independently generated codes from the initial 3 interview transcripts. These codes were compared for accuracy and consistency prior to continuing transcript coding for all interview transcripts. Codes were grouped to develop themes. Themes were again grouped to produce overarching themes. The development of themes and relationship between themes were clarified with review from all authors.

## Results

A total of 15 participants were invited and completed interviews, no participants declined to participate. Data saturation was assessed by two members of the study team (SBP, SO). Interviews had a mean duration of 30 minutes. Participants’ mean (standard deviation) age was 54.5 [14.7], 60% were white British, 80% were male, 60% had a sporting/exercise mechanism of injury. None of the participants were elite/professional athletes. All participants were experiencing their first ATR, with no participants reporting a previous ATR prior to the current injury. The mean time between ATR and interview was 8.9 (3.3) months.

Three main themes were identified from the data; the experience of injury and entering the healthcare system, the experience of non-surgical immobilisation, the rehabilitation journey. Across these themes, participants’ accounts showed that recovery was shaped not only by physical symptoms, but also by emotional responses to injury, behavioural adaptations during immobilisation and rehabilitation, and system-level factors such as service pressures, inconsistent information, and access to healthcare professional support. Themes and subthemes are presented below (Table 1), the coding tree is provided in S2 File.

**Table 1:** Main themes and sub-themes identified from participant interviews.

Main theme 1	Main theme 2	Main theme 3
The experience of injury and entering the healthcare system	The experience of non-surgical immobilisation	The rehabilitation journey
**Sub-theme 1.1:** The injury, presentation and receiving a diagnosis	**Sub-theme 2.1:** Patient experiences and perceptions of boot immobilisation	**Sub-theme 3.1:** Managing fear
**Sub-theme 1.2:** Treatment expectations and developing understanding	**Sub-theme 2.2:** Impact of immobilisation on the individual	**Sub-theme 3.2:** Returning to pre-injury health, sport and social activity

### Theme 1 – The experience of injury and entering the healthcare system

#### Sub-theme: The injury, the presentation and receiving a diagnosis.

This sub-theme describes how participants experienced ATR as a sudden event that created uncertainty about the seriousness of the injury and how to access appropriate care. Participants’ accounts suggested that the meaning they attached to the injury was shaped by the presence or absence of pain, previous Achilles symptoms, personal knowledge of anatomy, and the responses they received from healthcare services. For some, prompt recognition and diagnosis provided reassurance, whereas delays, uncertainty, or limited communication contributed to stress and concern about whether the injury was being managed appropriately.

**Injury mechanism.** Participants described the initial mechanism of ATR as sudden and unexpected. The sensation was frequently described as feeling like an impact from an external force and could occur with a high energy movement (e.g., sprinting) or a low energy movement (e.g., picking something up).

P02 - *I wondered if the goalposts had fallen down and hit me on the back of the left calf.*

P03 - *it felt as if my whole ankle had exploded. A bit like I imagine if I've been shot in my ankle*

**Pain following injury.** This sensation was accompanied by pain in some participants, while others reported no pain following the injury. The lack of immediate pain often complicated the recognition of injury severity and led to a misinterpretation of symptoms. This variability in symptom presentation often delayed care-seeking, as individuals downplayed their experiences or attributed them to more familiar diagnoses such as ankle sprains. Although factors such as a background of anatomy knowledge or insight from a family member or friend contributed to an accurate self-diagnosis.

P14 - *it wasn't painful, there was no pain with it, it was just the ability to not be able to use the foot.*

P11 - *the pain wasn't too bad, I was able to get in my car and drive home. I didn't go to the hospital thinking that it'll just get better*

P01 *- I knew immediately. It wasn't painful. I knew a little bit about the anatomy of it, compared to the normal, there was just floppy skin*

**Previous Achilles symptoms.** Many participants described experiencing long-standing Achilles discomfort, including pain, stiffness, or swelling, in the months or years prior to their ATR. These symptoms were often normalised, self-managed, or seen as part of an active lifestyle. The chronic nature of previous Achilles symptoms contributed to a perception that their pain may progress to an ATR.

P10 - *I've been suffering from tendinitis in both Achilles, predominantly my right one that's my non ruptured one, I've been to the GP about that, it was still under investigation when I ruptured my left Achilles.*

P15 - *from the pain that I'd probably had for 15 years, I thought that something was going to give at some point.*

**Entering the healthcare system.** A frequent experience reported by participants was extended waiting times in emergency departments. Several individuals described acute emergency departments as overwhelmed and under-resourced, which affected the quality of care they received. Participants reported that the busy environment limited the time for communication and there was consequent lack of reassurance about initial ATR management.

P10 - *They just can't have the time. I'd love to talk to an individual about it. I was like, I don't know if I was being scammed or anything, almost felt quite stressed at that stage*

**Alternate immobilisation experiences**. Service pressures in emergency departments also changed the initial treatment plan. If staff were not trained in providing the immobilisation boots, they were placed in a cast instead. This alternate approach required additional time and resources in comparison to routine boot immobilisation. Challenges to staff training and ATR awareness were not isolated to emergency departments. One participant reported an initial consultation in primary care where they received an uncertain diagnosis and they waited an additional 3 days before being immobilised.

*P04* - it was late in the day, so they hadn't got anybody that could fit the Vacoped boot at that point, so they said they were going to cast me.

*P08* - *I told her* (advanced nurse practitioner) *what happened.. She just said, see what it's like in a week. By mid-afternoon, she'd phoned me, best if they send me down for a scan, so that was a week after doing it.*

### Sub-theme: Treatment expectation and developing understanding

**Surgical expectations.** Participants’ experiences following ATR were shaped by initial expectations of surgical intervention, which evolved over time as they encountered established treatment pathways. Many participants reported an initial assumption that surgery would be the standard treatment for a ruptured tendon. This belief was based on their anatomical understanding of ATR and that surgery would be more resilient against re-rupture and appose tendon ends to improve healing.

P03 - *thinking of the mechanics of it, the boot is like sticking some super glue on. Yes, they merge, but they never quite merge in the same way. Whereas the view of surgery was that you've got a bit of a chain mail now fully attached. And it's going to take an awful lot for you to burst that*

**Introducing non-surgical management.** When non-surgical treatment was presented by healthcare professionals, participants described feelings of surprise. This was often received positively as non-surgical options avoided potential surgical risks. However, some scepticism remained regarding the plausibility of tendon healing without surgery.

P02:  *I was petrified that I'd be knocked out and stitched. So I was very pleased to be given a boot.*

P01 - *So the bit I was sceptical about was, is it possible to reattach two severed tendons that must be pulling apart just by putting an orthopaedic boot and putting the ends in the proximity of each other, seemed extremely unlikely to me.*

**Conflicting information.** Participants reported multiple causes of conflicting information. This occurred when consulting with healthcare professional and other members of the public. This created an impression that non-surgical management was inferior and a cost-saving alternative to surgical management.

P11 - *the thing that irked me was, them saying that we reserve this* (surgery) *for elite athletes. Which made me feel, if it's good enough for them, and if it makes them better quicker, then why is it not* (given) *to the public?*

P14 - *I was out at a dinner and I was in the boot and somebody said to me, ‘You want to get that sorted and get an operation because the boot don’t work’.*

P07 - *I was expecting it to be pinned together. But then there's also that worry about when you've been given the boot, is this the correct way or is this a cheap way*

### Theme 2 – The experience of non-surgical immobilisation

#### Sub-theme: Patient experiences and perceptions of boot immobilisation.

Participants described boot immobilisation as a central part of recovery that shaped their mobility, independence, comfort, hygiene, confidence, and sense of responsibility for self-management. Many participants viewed the boot positively, particularly when compared to casting. Although several critiques emerged regarding physical comfort, personal hygiene, ease of cleaning the boot and the personal responsibility of managing boot adjustments.

**Comparison to casting.** Participants who were initially provided a non-weightbearing cast often reported difficulty with balance, discomfort, and limited mobility. The transition to a weightbearing boot enabled greater mobility and independence.

P06 - *It was brilliant the boot, because I could have a relatively normal life instead of being in a cast. I was able to put weight on my bad leg… I can actually not fall over.*

**Immobilisation protocol deviations.** Despite its advantages, personal hygiene and maintaining cleanliness of the boot was a challenge during immobilisation. This occurred alongside irritation from the liner of the boot which required regular adjustments. These issues caused participants to deviate from the prescribed immobilisation protocol. One participant reported that this was driven by discomfort during the night time. Another participant reported weight bearing through the affected limb to maintain safety when washing.

P09 - *I washed it every two days because the smell. I still think about the smell*

P12 - *sometimes they got too uncomfortable at night so I used to take it out.*

P10 - *I put a little bit of weight on the floor. Because automatically, you want to balance yourself* (when showering)

**Immobilisation boot self-adjustments.** Participants reported that they were required to self-adjust the boot at regular intervals during the immobilisation period. This responsibility led to confusion, errors, and heightened anxiety but was seen as necessary due to service constraints.

P03 - *When it came to the first adjustment… there are two adjusting points… honestly… you’re being told so much in that first session… I just couldn’t remember.*

P10 - *I may have changed the slope to something it shouldn’t have been… I felt like a bit of a plonker.*

**Self-management during immobilisation**. A key concern was the fear of long-term consequences from an incorrect boot adjustment. Some participants reported feeling isolated when left with self-management and this caused a sense of abandonment from the health service. To manage this, participants reported contacting the physiotherapy department but they were not able to reach the necessary healthcare professional.

P07 - You’re told how to use it, then we’ll see you again in eight weeks… there’s that feeling of abandonment

P10 - I remember wanting to ring the hospital, but the number for the physio department doesn't get answered

### Sub-theme: Impact of immobilisation on the individual

Participants consistently highlighted the personal impacts of boot immobilisation, extending beyond physical recovery. Psychological strain, perceived physical decline, altered working life, and secondary musculoskeletal issues emerged as key concerns during the immobilisation period.

**Impact on mental wellbeing.** Many participants described a significant psychological challenge associated with immobilisation. In participants who were active prior to injury, exercise was a management strategy for mental wellbeing and this was removed by immobilisation. The inability to complete pre-injury exercise created a perpetuating cycle of declining mental wellbeing.

P10 **–** *My mental health did drop dramatically… Exercise helps my mental health massively. But when your mental health is low, to force yourself out to do stuff is difficult*

**Weight gain.** The impact of immobilisation on reducing activity levels caused participants to gain weight. This could occur after a pre-injury increase in activity which was motivated by the losing weight.

P02 - *I was eating a lot, put a lot of weight on... it was a huge job to get the weight off. I put on a stone and a half*

P10 - *I did put on a stone or so since the rupture... just sitting down and snacking*

**Returning to work.** The influence of immobilisation on physical and mental wellbeing also shaped participants working lives. The impact of ATR on the individual’s health was reported as a unique life event by one participant. Participants who were highly motivated to return to work acknowledged the challenge of long rehabilitation timeframes.

P11 - *I didn't go to work for two or three weeks. And I felt really down. I think it's the first time in my life… I ever felt mentally down*

P04 - *Even if you're like me, incredibly positive and desperate to work... it's a longer process than you ever imagine*

**Musculoskeletal pain development.** The immobilisation boot impacted the participants walking. This caused additional musculoskeletal issues which could further limit mobility.

P12 - *I think that* (immobilisation boot) *disturbed the pelvis in a certain way and has caused the sciatic nerve to become aggravated*

P03 - *Pain in my back walking was astronomical… it really was driving me nuts*

### Theme 3 – The rehabilitation journey

Theme 3 describes rehabilitation as a process of rebuilding confidence and trust in the injured tendon, rather than simply progressing through physical recovery milestones. Participants’ accounts showed that the transition out of immobilisation created uncertainty about whether the tendon was strong enough to tolerate everyday movement, rehabilitation exercises, and return to sport. Fear of re-rupture, reduced confidence, ongoing pain, and limited psychological support shaped how participants approached activity, made decisions about risk, and judged their readiness to return to pre-injury roles and sporting identities.

### Sub-theme: Managing fear

Fear was a persistent component of participants’ rehabilitation journeys following ATR, shaping how they interpreted pain, judged tendon vulnerability, approached exercise, and made decisions about returning to activity or sport. As participants transitioned from the immobilisation boot to rehabilitation, many described heightened vulnerability, anxiety about re-injury, and psychological barriers that influenced both everyday activity and long-term return to sport.

**Immobilisation boot removal.** The removal of the boot marked a turning point in rehabilitation but also the beginning of anxiety around movement for many. The immobilisation boot provided a sense of security and allowed relatively normal daily activities. Participants expressed feeling that the tendon was fragile and they were unsure about the safety of their movement.

P01 - *As soon as I took the boot off and I put the tiniest bit of pressure on that, I knew that it was vulnerable and sore… the whole brain was screaming, don't do this*

P04 - *I felt fragile, nervous, that I was going to just put the wrong step forward or attempt the stairs on the wrong angle* (and) *re-rupture*

Several participants highlighted a desire for imaging to provide visual confirmation that healing had occurred. This was motivated by the non-surgical strategy providing no signs of an intervention in comparison to a surgical incision.

P05 - *If it was a scar or something, you know an operation, you could see it healing and mending... but there was nothing to see. Nothing to feel… imaging would have reassured the professional to say, ‘No, you're okay”*

**Nature of ATR mechanism.** The unpredictable nature of rupture and potential re-rupture led participants to adopt cautious behaviours or avoid activities altogether. Returning to sport emerged as a considerable psychological hurdle. Some participants reported avoiding former sports completely, not due to lack of physical ability but due to a lack of confidence in their tendon and fear of repeating the injury

P12 - *You still have it in your mind that it happened so unexpectedly so it can happen again*

P07 *- I think it's not worth it, in all honesty. I think for me, the badminton rackets hung up.*

P13 - *My goal was to go and play badminton. I haven't returned... I just don't feel comfortable and confident that the Achilles is going to stand up to it*

**Mental health support.** Participants noted that this fear of returning to activity was compounded by a perceived absence of mental health support within the rehabilitation process. Participants acknowledged that themselves and healthcare professionals separated the physical and psychological aspects of their rehabilitation.

P06 - *It* (being active) *was a mental block… and I kind of link it to mental health … I don't think that was accounted for in some of the physio sessions… Not interested in that side of things*

### Sub-theme: Returning to pre-injury health, sport and social activity

**Previous sport engagement.** Participants highlighted the long and complex path toward regaining their pre-injury physical health and identity. Motivation played a critical role in participants rehabilitation experiences. Some participants felt that their previous sporting experience supported their engagement with an active exercise approach to rehabilitation. However, others acknowledged struggling with the monotony of rehabilitation and the lack of enjoyment in comparison to sport.

P02- *because I've played loads of sport all my life, I'm very, very used to all that. So I was just lapping up the exercise*

P10 – *It* (rehabilitation) *just felt so monotonous, when I want to be out running*

P09 - *Even now, I can't even do* (calf raises) *like properly… I would rather play volleyball*

**Long term Achilles pain.** Ongoing pain was cited as a key barrier to progress. Participants described localized pain, stiffness, and fatigue in the affected limb, reducing their ability to engage fully with previous levels of activity.

P12 - After a while it just gets sore…or if I'm not doing anything for a while then it can get sore as well. I'd say it's like a 6 out of 10 pain

**Social impact of returning to sport.** Participants described how returning to the social elements of sport and recapturing their identity was a motivator to engage with rehabilitation. Other participants reported that their inability to return to sport had socially isolated them.

P08 - *I had a focus that I needed to do that, to be able to get back to how I was… to be able to live my life*

P13 - *I’ve missed the social side of playing badminton… we’d go to the bar or go to the pub… it’s not the same just turning up afterwards*

**Healthcare professional advice.** Some participants reflected on receiving discouragement from healthcare professionals about returning to previous sporting levels. This influenced decisions to avoid certain activities, sometimes permanently.

P13 - *They've* (physiotherapist) *said that I should not consider playing* (badminton) *because of the amount of strain that's put on* (the Achilles tendon)*… they've suggested I could continue playing golf*

## Discussion

This study provides novel insights into the experiences of individuals undergoing non-surgical management for ATR within the NHS. Three main themes were identified; The experience of injury and entering the healthcare system, The experience of non-surgical immobilisation and The rehabilitation journey. The findings highlight the diverse range of injury presentations and the varied adaptations to acute management, as well as the multifaceted factors that influence adherence to immobilisation protocols and rehabilitation.

### The experience of injury and entering the healthcare system

This study provides important insights for clinicians, highlighting that ATR presents with diverse mechanisms and presentations. Consistent with previous descriptions [17], many participants reported the sensation of being struck in the Achilles region. However, this sensation could occur without significant pain and may result from a low-energy mechanism. Some individuals described a preceding history of Achilles symptoms or initially suspected an alternative diagnosis, such as an ankle inversion injury. This variation in presentation reinforces the need for appropriate training for healthcare professionals completing assessments, as misdiagnosis of acute ATR can lead to delayed treatment, increased disability, and higher healthcare costs [18].

The impact of limited healthcare professional training and system pressures such as understaffing on the initial management of ATR was also reported by participants. This caused delayed diagnosis, patient apprehension and alternate initial management approaches such as casting. Casting was used despite the recognised use of an established immobilisation protocol [11]. This emphasises the importance of training and service improvements to enhance the quality and consistency of care. Future studies should consider the impact of modified initial immobilisation procedures when evaluating the outcomes of ATR.

There has been a transition to non-surgical approaches for ATR management with 75% of individuals managed non-surgically in the UK [19]. Despite this, participants had an expectation that acute ATR management would be surgical repair. There was a perception of a lower re-rupture rate with surgical management and that non-surgical approaches were a cost-saving alternative. This perspective reflects multiple reviews and investigations published on ATR management [20–24]. However, several participants were relieved at avoiding the potential complications of surgery which also reflects the increased complication rate reported with surgical management [21–24]. Patients should receive clear, evidence informed information about current treatments and their respective risks and benefits. However, patient uncertainty could be seen to reflect the ongoing debate on the most effective management strategies for ATR.

### The experience of non-surgical immobilisation

The early weight bearing provided by the LAMP was well received by participants, with participants appreciating the increased mobility it afforded compared to non-weightbearing casting. This reflects evidence that early functional rehabilitation can enhance patient satisfaction and functional outcomes [25]. However, practical challenges of personal and boot hygiene were highlighted. Personal hygiene practices are important in monitoring skin health during immobilisation as skin complaints are a complication reported in both surgical and non-surgical management [26]. One participant reported weightbearing without the immobilisation boot to prevent falling during showering. These modifications to the immobilisation protocol may impact tendon integrity and contribute to tendon elongation and should be monitored when evaluating immobilisation protocols. Healthcare professionals should provide guidance on hygiene practices to mitigate compromises to tendon healing during the immobilisation phase.

Participants were asked to complete adjustments of the immobilisation boot position independent of a healthcare professional. This independence caused incorrect boot adjustments for some and was seen as abandonment by one participant. This was increased by the inability to contact a healthcare professional who was engaged with their care. If the boot position is incorrectly adjusted patients would experience increased dorsiflexion too early in their recovery, this may expose patients to a higher risk of elongating the tendon. As elongation mainly occurs during the initial months post ATR, and elongation is associated with heel rise limitations, further support such as written or video guidance should be given to ensure accurate boot adjustments [26,27].

Post-ATR immobilisation was reported to have a detrimental impact on participants physical and mental health. Particularly those for whom exercise had previously been an important coping mechanism for stress and wellbeing. The abrupt loss of physical activity and social engagement was perceived to compound psychological strain, creating a cycle that hindered motivation for rehabilitation. Participants also reported gaining weight during immobilisation due to inactivity and dietary changes. This presents a health risk for the ATR population that are already typically overweight (Body Mass Index>25) [28]. Weight gain may further impede functional performance during rehabilitation exercises as a one unit increase in BMI results in a three percent decrease in calf raise repetitions [29]. As the immobilisation period was reported to be detrimental to overall wellbeing, immobilisation protocols should account for this when considering protocol duration.

Participants also reported secondary musculoskeletal complaints, including back pain and sciatic nerve irritation, likely linked to altered gait mechanics while using the boot. Altered movement mechanics has been found previously to cause additional musculoskeletal pain sites [12]. Such issues risk prolonging disability and highlight the potential benefits of shoes raises on the other limb.

### The rehabilitation journey

Consistent with previous studies, participants described a strong fear of movement (kinesiophobia) and a persistent fear of re-rupture, which influenced their willingness to load the tendon, engage with rehabilitation exercises, and return to pre-injury sports [12,30,31]. The perceived unpredictable nature of ATR was described as heightening this fear, with participants feeling anxious about the safety of everyday movements once the immobilisation boot was removed. Interestingly, the absence of a visible marker of healing, such as a surgical scar, exacerbated uncertainty for those managed non-surgically. Many participants expressed a desire for imaging to provide objective evidence that the tendon had healed sufficiently to tolerate loading.

Participants withdrew from previous sports due to a sense of inadequate physical readiness and fear of re-rupture. This is consistent with previous qualitative studies showing that fear is a primary factor limiting return to sport after ATR [12,31]. This impacted the quality of life of participants as it isolated them socially and changed them from their pre-injury identity. Participants also described how ongoing pain, stiffness and fatigue continued to limit their ability to return to sport, even when motivated to do so. It is unclear if interventions are required to address the psychological components influencing fear of re-rupture, or sufficient physical rehabilitation consequently reduces fear of re-rupture.

Participants’ accounts raise questions about the role of healthcare professionals in either supporting or inadvertently limiting return to activity. Some described physiotherapists actively discouraging them from returning to previous sports due to perceived re-injury risks. Similar findings have been identified in individuals following anterior cruciate ligament rupture, where risk-averse messaging from clinicians influenced patients’ perceptions of returning to sport [32]. This reflects a tension between clinical caution and patients’ goals to regain their prior activity levels and social connections. Future research should explore how clinicians navigate this balance and the potential impact of risk-averse messaging on patients’ confidence and activity levels.

### Strengths and limitations

This study provides a novel insight on the early stages of recovery as participants were recruited within 12 months of injury. This contrasts with previous studies that have often focused on longer-term experiences, with participants recalling events years after ATR [12]. By focusing on this earlier timeframe, the study captures more immediate perceptions of the initial injury and recovery.

The percentage of male participants was consistent with previous ATR investigations in the UK and the sample consisted of range of ethnicities [9]. The age of participants was higher than in previous qualitative investigations that focused on return to sport post ATR [12,31]. This difference likely reflects the decision in the present study not to exclude older adults, who are experiencing an increasing incidence of ATR [33]. As the average age of ATR rises, it is essential that the older cohort perspectives are understood and reflected in clinical decision-making.

Participants were recruited from a single NHS ATR clinic, which may limit the transferability of findings to other healthcare settings, particularly those outside the UK or with different immobilisation protocols. Furthermore, the study included only individuals treated non-surgically and may not represent surgically managed patients. Future research could compare experiences across treatment approaches to understand how clinical pathways influence recovery experiences.

Long term functional deficits have been reported following ATR [4–6]. Due to the qualitative nature of this study, the relationship between reported fear of re-injury and objective measures of Achilles performance are unknown. Triangulating qualitative findings with functional outcomes in future studies would provide an understanding of how patient experience aligns with functional capacity.

## Conclusion

This study provides new insights into the lived experiences of individuals recovering from ATR managed non-surgically within the NHS. By exploring the patient journey from injury through rehabilitation, the findings highlight the complex and wide-ranging factors that may influence long term recovery.

## Supporting information

S1 File Interview topic guide. (DOCX)

S2 File Coding tree. (DOCX)
