# Supplementary material for: The experience of individuals following non-surgical management of Achilles tendon rupture in the United Kingdom – a qualitative study
Source: PLoS One. 2026 Jun 29;21(6):e0352761. doi: 10.1371/journal.pone.0352761 (PMC13313361; doi:10.1371/journal.pone.0352761)
Supplement: S1 File — (DOCX) [file pone.0352761.s001.docx]

**Interview Topic Guide**

**Background of patients Achilles tendon rupture**

Can you start by telling me about your rupture, how it happened and the management you’ve had so far?

Prompt

- When was your rupture date?
- How did it happen?
- Did you have any symptoms prior to this?
- Did you have previous Achilles pain ever? If yes – what treatments did you receive? What diagnoses did you receive?

What immediate diagnosis and advice did you receive?

Prompt

- Were you happy with this advice?

Was it clear, did you understand it?

- What further advice would you have wanted?
- What do you understand by a “rupture”
- Was diagnostic imaging use (Ultrasound or MRI) to confirm diagnosis?

Would you have preferred non-surgical or surgical management of your Achilles rupture?

Prompt

- Were both options offered?
- Was an opinion given about the differences between the two?
- Is surgical, why? If non-surgical, why?
- Did the clinician explain how a rupture would heal without surgery? How did you think a rupture would heal without surgery.

**Achilles Tendon Immobilisation**

How did you find the boot immobilisation?

Prompt

- Was it comfortable?
- Did it effect your sleep?
- Were you allowed to take the boot off to shower etc?

What advice were you given regarding the boot and immobilisation period?

Prompt

- Was it clear what you were supposed to do e.g boot adjustment, skin management?

- Did you feel happy with this advice?

- What other advice would you have liked?

- Did you follow the advice regarding the boot?

Prompt

- If not, why not?

What would have made the experience of wearing the boot easier?

Would you have preferred to have a longer or shorter immobilisation time?

Would you be willing to consider starting rehabilitation exercises whilst using the boot?

Prompt

- If not, why?

Would you have liked more clinical reviews

Prompt

- If yes, why? Would you have like them in person or virtually/telephone
- If no, why?

**Post Achilles Tendon Boot Removal**

How did you feel once the boot was removed?

Prompt

- Were you confident?
- Did you have any anxieties?
- Would you have like further imaging e.g ultrasound?
- Did you remove the boot at home first? If so, why?

How did you feel about exercising once the boot had been removed?

What advice was you given about exercise immediately after boot removal?

Prompt

-Which exercises were you given? How many reps/set of exercises? How frequent?

-Was information verbal or written?

-Was pain during exercise discussed?

-What about guidance with walking (distance and frequency)?

- What advice/discussions did you have about footwear? What did you actually wear?

When were you referred to physiotherapy?

What was the length of time between physiotherapy referral and your initial appointment?

When did you start physiotherapy?

What has physiotherapy rehabilitation consisted of (strength, range of motion, fitness)?

How do you feel about the content of your rehabilitation?

Do you feel you have a good understanding of why you are doing rehabilitation?

Have you / Are you achieving what you would like to during rehabilitation?

Prompt

- What have your goals been and has this been discussed with your physio (walking, hobbies, family duties)
- what did you want to get back to?
- Are you back to everything?
- Have you had to make lifestyle adjustments?

What are your expectations for rehabilitation?

Prompt

- Timeframe, content, frequency

What would an optimal rehabilitation programme look like to you?

Prompt

- A ‘dream’ programme without financial or time restrictions
- Content, frequency, contact with a professional, imaging, strength tests, step counts, boot removal early, starting exercise whilst in the boot, Exercising the other limb

**Barriers to Rehabilitation**

What have been the main barriers to engaging with your rehabilitation?

Prompt

- Patient time, clinician time/appointments, pain, anxieties
- Work, transport, social responsibilities

**Facilitators to Rehabilitation**

What has made you rehabilitation process easier?

Prompt

- Location, equipment, appointment times

What has motivated you to engage with rehabilitation?

What would make you rehabilitation process easier?

**Health and Wellbeing**

What has been the impact of your Achilles tendon rupture on you?

Prompt

- Your family, your work, your hobbies/activities

Has your overall physical and mental health been affected by your Achilles tendon rupture?

How has this made you feel?

What advice would you give to someone who has recently experienced an Achilles tendon rupture?

If you have any other health conditions, how do you feel these effect or are affected by your Achilles tendon rupture?

If you would change anything following your Achilles tendon rupture, what would you change?
