# Supplementary material for: The experience of individuals following non-surgical management of Achilles tendon rupture in the United Kingdom – a qualitative study
Source: PLoS One. 2026 Jun 29;21(6):e0352761. doi: 10.1371/journal.pone.0352761 (PMC13313361; doi:10.1371/journal.pone.0352761)
Supplement: S2 File — (DOCX) [file pone.0352761.s002.docx]

| The experience of injury and entering the healthcare system | The injury, the presentation and receiving a diagnosis | Inactive prior to injury |
| --- | --- | --- |
|  |  | Painless mechanism of injury |
|  |  | Awareness of ATR after MOI |
|  |  | Achilles symptoms prior to ATR |
|  |  | Able to walk post MOI |
|  |  | Pain following injury |
|  |  | Psychological perspective |
|  |  | Previous Achilles treatments |
|  |  | Previous exercise experience and rehab |
|  |  | A&E waiting times |
|  |  | Diagnostic tests in A&E |
|  |  | Urgent care ultrasound diagnosis |
|  |  | Urgent care triage |
|  |  | Urgent care initial plaster |
|  |  | Initial medical triage |
|  |  | Injury to immobilisation time |
|  |  | Healthcare professional diagnosis description |
|  | Treatment expectation and developing understanding | Preference for surgical management |
|  |  | Perceived low re-rupture with surgery |
|  |  | Surgical repair expectation |
|  |  | Sceptical about non-surgical management |
|  |  | Satisfaction with non-surgical management |
| Abbreviations: ATR=Achilles tendon rupture, MOI=Mechanism of Injury, A&E=Accident and Emergency | | |

| The experience of non-surgical immobilisation | The evaluation of boot immobilisation | Dynamised Immobilisation boot evaluation |
| --- | --- | --- |
|  |  | Dynamised Immobilisation self-adjustment advice |
|  |  | Incorrect Dynamised Immobilisation boot adjustment |
|  |  | Removing boot heel during sleep |
|  |  | Night time mobilisation with boot |
|  |  | Graded removal of immobilisation boot |
|  |  | Self-management with boot |
|  | Impact of immobilisation on the individual | Immobilisation duration |
|  |  | Extended immobilisation period |
|  |  | Weight gain during immobilisation |
|  |  | Difficulty washing during immobilisation |
|  |  | Loss of strength during immobilisation |
|  |  | Overall health effects of immobilisation |
|  |  | Psychological impact of immobilisation |
|  |  | Impact of health comorbidities |
|  |  | Other health comorbidities limiting activity |

| The rehabilitation journey | Managing Fear | Fear of reinjury on boot removal |
| --- | --- | --- |
|  |  | Anxieties on boot removal |
|  |  | Fear of reinjury during rehab |
|  |  | Fear of re-rupture |
|  |  | Anxieties about contralateral rupture |
|  |  | Exercise fear avoidance |
|  |  | Fear of surgical management |
|  | Returning to pre-injury health and social activity | Psychological perspective |
|  |  | Motivation to rehab |
|  |  | Lifestyle barriers to rehabilitation |
|  |  | Psychological benefits of exercise |
|  |  | Previous exercise experience and rehab |
|  |  | Previous Achilles treatments |
|  |  | Pain and rehab exercises |
|  |  | Difficulty washing after immobilisation |
|  |  | Rehabilitation goals |
|  |  | Returning to goals |
|  |  | Returning to sport |
|  |  | Long term reduced activity |
|  |  | Long term strength deficit |
|  |  | Long term symptoms |
|  |  | Satisfaction with long term outcome |
|  |  | Walking after immobilisation period |
|  |  | Impact of health comorbidities |
|  |  | Other health comorbidities limiting activity |
