# Supplementary material for: The experience of individuals following non-surgical management of Achilles tendon rupture in the United Kingdom – a qualitative study
Source: PLoS One. 2026 Jun 29;21(6):e0352761. doi: 10.1371/journal.pone.0352761 (PMC13313361; doi:10.1371/journal.pone.0352761)
Supplement: Attachment — Submitted filename: Response to Reviewers.docx [file pone.0352761.s004.docx]

Thank you for taking the time to review our manuscript. All reviewer comments have been accepted and appropriate modifications made. Please see below for a point-by-point response to feedback

Line numbers provided in this response refer to the clean manuscript file.

|  | **Journal Requirements / Editor** | **Response** |
| --- | --- | --- |
| 1 | Please ensure that your manuscript meets PLOS ONE's style requirements, including those for file naming. The PLOS ONE style templates can be found at  https://journals.plos.org/plosone/s/file?id=wjVg/PLOSOne_formatting_sample_main_body.pdf and  https://journals.plos.org/plosone/s/file?id=ba62/PLOSOne_formatting_sample_title_authors_affiliations.pdf | We have reviewed the manuscript against the PLOS ONE style requirements, including manuscript formatting and file naming requirements. The manuscript and supporting files have been amended where required to comply with PLOS ONE formatting guidance. |
| 2 | Thank you for stating the following in the Acknowledgments Section of your manuscript:  “This project was completed as part of a Future 100 PhD Studentship at the University of Leicester”  We note that you have provided funding information that is not currently declared in your Funding Statement. However, funding information should not appear in the Acknowledgments section or other areas of your manuscript. We will only publish funding information present in the Funding Statement section of the online submission form.  Please remove any funding-related text from the manuscript and let us know how you would like to update your Funding Statement. Currently, your Funding Statement reads as follows:  “This project was completed as part of a Future 100 PhD Studentship at the University of Leicester”  Please include your amended statements within your cover letter; we will change the online submission form on your behalf. | Thank you for highlighting this issue. Funding-related text has been removed from the manuscript where required. We would like the Funding Statement in the online submission form to read: “This project was completed as part of a Future 100 PhD Studentship at the University of Leicester.” |
| 3 | Thank you for stating the following financial disclosure:  “This project was completed as part of a Future 100 PhD Studentship at the University of Leicester”  Please state what role the funders took in the study. If the funders had no role, please state: "The funders had no role in study design, data collection and analysis, decision to publish, or preparation of the manuscript."  If this statement is not correct you must amend it as needed.  Please include this amended Role of Funder statement in your cover letter; we will change the online submission form on your behalf. | Thank you for this comment. We confirm that the Role of Funder statement should read: “The funders had no role in study design, data collection and analysis, decision to publish, or preparation of the manuscript.” |
| 4 | Thank you for stating the following financial disclosure:  “This project was completed as part of a Future 100 PhD Studentship at the University of Leicester”  We note that one or more of the authors is affiliated with the funding organization, indicating the funder may have had some role in the design, data collection, analysis or preparation of your manuscript for publication; in other words, the funder played an indirect role through the participation of the co-authors. If the funding organization did not play a role in the study design, data collection and analysis, decision to publish, or preparation of the manuscript and only provided financial support in the form of authors' salaries and/or research materials, please do the following:  Review your statements relating to the author contributions, and ensure you have specifically and accurately indicated the role(s) that these authors had in your study. These amendments should be made in the online form.  Confirm in your cover letter that you agree with the following statement, and we will change the online submission form on your behalf:  “The funder provided support in the form of salaries for authors [insert relevant initials], but did not have any additional role in the study design, data collection and analysis, decision to publish, or preparation of the manuscript. The specific roles of these authors are articulated in the ‘author contributions’ section. | Thank you for this clarification. We have reviewed the author contribution statements and confirm that the roles of each author are accurately stated. We agree with the following statement for the online submission form: “The funder provided support in the form of salary/studentship support for author SBP, but did not have any additional role in the study design, data collection and analysis, decision to publish, or preparation of the manuscript. The specific role of this author is articulated in the ‘author contributions’ section.” |
| 5 | In the online submission form you indicate that your data is not available for proprietary reasons and have provided a contact point for accessing this data. Please note that your current contact point is a co-author on this manuscript. According to our Data Policy, the contact point must not be an author on the manuscript and must be an institutional contact, ideally not an individual. Please revise your data statement to a non-author institutional point of contact, such as a data access or ethics committee, and send this to us via return email. Please also include contact information for the third party organization, and please include the full citation of where the data can be found. | Thank you for this comment. The Data Availability Statement has been revised so that requests are directed to a non-author institutional point of contact rather than to a manuscript author. The revised statement is: “The datasets generated and analysed during the current study are not publicly available due to the confidentiality of qualitative interview data and the conditions of ethical approval. Reasonable requests for anonymised excerpts may be considered by the University of Leicester Research Governance Office, subject to institutional approval and data sharing governance.” |
| 6 | Your ethics statement should only appear in the Methods section of your manuscript. If your ethics statement is written in any section besides the Methods, please delete it from any other section. | Thank you for this comment. The ethics statement has been retained in the Methods section only. The duplicate ethics statement in the Declarations section has been removed. |
| 7 | Please include captions for your Supporting Information files at the end of your manuscript, and update any in-text citations to match accordingly. Please see our Supporting Information guidelines for more information: http://journals.plos.org/plosone/s/supporting-information. | Thank you for this comment. Captions for the Supporting Information files have been added at the end of the manuscript under the heading “Supporting information”. The in-text citations have also been updated to match the Supporting Information file names: the interview topic guide is cited as S1 File and the coding tree is cited as S2 File. |
| 8 | If the reviewer comments include a recommendation to cite specific previously published works, please review and evaluate these publications to determine whether they are relevant and should be cited. There is no requirement to cite these works unless the editor has indicated otherwise. | Thank you for this guidance. We will review and evaluate any reviewer-recommended publications to determine whether they are relevant and should be cited. No additional references have been added at this stage in response to the Journal Requirements / Editor comments. |
| 9 | Please review your reference list to ensure that it is complete and correct. If you have cited papers that have been retracted, please include the rationale for doing so in the manuscript text, or remove these references and replace them with relevant current references. Any changes to the reference list should be mentioned in the rebuttal letter that accompanies your revised manuscript. If you need to cite a retracted article, indicate the article’s retracted status in the References list and also include a citation and full reference for the retraction notice. | Thank you for this comment. The reference list has been reviewed for completeness and accuracy. Reference 15 has been corrected to include the complete PLOS ONE article number. We have not knowingly cited any retracted articles. Any changes to the reference list have been noted in this response. |
|  | **Reviewer 1** |  |
| 1 | The term “stakeholders” is vague. Please specify their roles (e.g. patients, clinicians, physiotherapists) and stage of involvement (development, piloting, refinement). | Thank you for this comment. We have clarified the term “stakeholders” in the Methods section and Patient and public involvement section. The manuscript now specifies that the stakeholders were patient stakeholders with lived experience of ATR, and that they were involved during the development and refinement stages of the interview topic guide by reviewing the relevance, clarity, and acceptability of the proposed interview questions. (lines 104 and 602) |
| 2 | Please provide the full name of the COREQ guideline at first mention. | Thank you for this comment. The full name of the COREQ guideline has now been provided at first mention in the Methods section as “Consolidated Criteria for Reporting Qualitative Research (COREQ)”. (line 117) |
| 3 | The study population requires clearer definition. It is unclear whether participants differed in injury duration, activity level (athlete vs non-athlete), or whether injuries were first-time or recurrent, all of which may influence experiences. | Thank you for this comment. We have revised the Results section to provide a clearer description of the study population. We have clarified that none of the participants were elite/professional athletes, all participants were experiencing their first ATR, and no participants had experienced a previous ATR prior to the current injury. The manuscript already reports the mean time between ATR and interview, which describes the injury duration within the sample. (line 156) |
| 4 | - Line 159: please check for a spelling error. | Thank you for identifying this. We have checked and corrected the wording/capitalisation error in the list of theme names. |
| 5 | Consider labelling tables more clearly to distinguish between themes and their descriptions | Thank you for this comment. We have revised Table 1 to label the main themes and sub-themes more clearly. Each column now identifies a main theme, and each associated sub-theme is labelled numerically beneath the relevant main theme. (line 169) |
| 6 | Please specify the qualitative design (e.g. thematic analysis, phenomenology). | Thank you for this comment. We have revised the opening sentence of the Materials and methods section to specify that the study used a qualitative design with reflexive thematic analysis of in-person, one-to-one, semi-structured interviews. This clarification distinguishes the study design from other qualitative approaches such as phenomenology. (line 99) |
| 7 | - The concept of “lived experiences” is currently broad. Clarify key domains such as pain/function, psychological impact (e.g. fear, confidence), and interaction with healthcare services. | Thank you for this comment. We have revised the study aim in the Introduction to clarify the key domains included within “lived experiences”. The aim now specifies that the study focused on pain and function, psychological responses such as fear and confidence, interactions with healthcare services, experiences of immobilisation and rehabilitation, and attempts at returning to sport. (line 90) |
| 8 | Themes would benefit from greater interpretive depth, incorporating emotional responses, behavioural aspects (e.g. adherence, coping), and system-level influences. | Thank you for this comment. We have revised the Results section to provide greater interpretive framing of the themes. Specifically, we added a summary statement clarifying that participants’ recovery experiences were shaped not only by physical symptoms, but also by emotional responses to injury, behavioural adaptations during immobilisation and rehabilitation, and system-level factors such as service pressures, inconsistent information, and access to healthcare professional support. (line 162) |
| 9 | The study aim includes return to sport, but this is not clearly reflected as a theme or subtheme. | Thank you for this comment. We have revised the Theme 3 sub-theme label to make the return to sport component more explicit. The sub-theme previously titled “Returning to pre-injury health and social activity” has been amended to “Returning to pre-injury health, sport and social activity” in both Table 1 and the corresponding Results sub-heading. This clarifies that return to sport was represented within Theme 3. (lines 169 and 399) |
| 10 | Theme 1 (Subtheme 1): Currently descriptive; would benefit from a more patient-centred, interpretive approach, moving from “what happened” to “how it was experienced”. | Thank you for this comment. We have revised Theme 1, Sub-theme 1 to provide a more patient-centred and interpretive framing. Specifically, we added an opening paragraph that explains how participants experienced ATR as a sudden and disruptive event, how uncertainty was shaped by pain, previous Achilles symptoms, personal knowledge, and healthcare responses, and how delays or limited communication contributed to stress and concern about whether the injury was being managed appropriately. (line 176) |
| 11 | - Theme 2: The subtheme appears procedural. Consider focusing on patient experiences and impacts, and replacing “evaluation” with experience- or perception-based terminology | Thank you for this comment. We have revised the Theme 2 sub-theme label to use more patient-centred, experience-based terminology. The sub-theme previously titled “The evaluation of boot immobilisation” has been renamed “Patient experiences and perceptions of boot immobilisation” in both Table 1 and the corresponding Results sub-heading. We have also revised the opening sentence of the sub-theme to emphasise how boot immobilisation shaped participants’ mobility, independence, comfort, hygiene, confidence, and responsibility for self-management. (lines 273) |
| 12 | Theme 3: While relevant, the presentation is somewhat descriptive and technical. Greater emphasis on patient experience and psychological processes is recommended. | Thank you for this comment. We have revised Theme 3 to provide greater emphasis on patient experience and psychological processes during rehabilitation. Specifically, we added an interpretive opening paragraph framing rehabilitation as a process of rebuilding confidence and trust in the injured tendon, rather than only progressing through physical recovery milestones. We also clarified how fear of re-rupture, reduced confidence, ongoing pain, and limited psychological support shaped participants’ decisions about activity, perceived risk, and readiness to return to pre-injury roles and sporting identities. (lines 350) |
|  | **Reviewer 2** |  |
| 1 | revisions required include: expanded reflexivity statement addressing the lead author's professional positionality | Thank you for this comment. We have expanded the reflexivity statement in the Materials and methods section to address the lead author’s professional positionality. The revised text now clarifies that the lead author’s background as a physiotherapist and PhD researcher provided familiarity with ATR management and rehabilitation pathways, which supported the development of clinically relevant interview prompts. We have also clarified that reflexivity was supported through the use of a reflexive diary during data collection and analysis, and through discussion of emerging codes and themes with the wider authorship team. (line 111) |
| 2 | clarification of the saturation assessment timeline; | Thank you for this comment. We have clarified the saturation assessment timeline in the Participants subsection. The revised text now states that saturation was assessed during the recruitment period, after the initial 10 interviews had been completed and transcribed, and was reviewed alongside ongoing recruitment until data saturation was reached or the upper limit of 20 participants was met. (line 131) |
| 3 | correction of plural possessive apostrophe errors throughout | Thank you for this comment. We have reviewed the manuscript for plural possessive apostrophe errors and corrected these throughout. |
| 4 | expanded specificity in the conclusion regarding the form of guidance and support recommended. | Thank you for this comment. We have revised the abstract conclusion to provide greater specificity regarding the form of guidance and support recommended. The revised sentence now highlights the need for clearer guidance on boot use, weightbearing, and rehabilitation progression, alongside consistent healthcare professional support to optimise outcomes. (line 52) |
